# In-vivo Studies on Sustained Release Systems for Delivering Antimycotics. A Systematic Review focused on Oral Candidiasis treatment

**DOI:** 10.4317/jced.63305

**Published:** 2025-10-17

**Authors:** Luis Alberto Gaitán-Cepeda, Maira Estrella Huerta-Reyes, Luis Octavio Sánchez-Vargas, María del Carmen Villanueva-Vilchis

**Affiliations:** 1 Departamento de Medicina y Patología Oral y Maxilofacial, División de Estudios de Postgrado e Investigación, Facultad de Odontología, Universidad Nacional Autónoma de México. México; 2 Unidad de Investigación Médica en Enfermedades Nefrológicas, Hospital de Especialidades “Dr. Bernardo Sepúlveda Gutiérrez”, Centro Médico Nacional Siglo XXI, Instituto Mexicano del Seguro Social. México; 3 Laboratorio de Bioquímica y Microbiología Oral, Facultad de Estomatología, Universidad Autónoma de San Luis Potosí. San Luis Potosí, Mexico; 4 Departamento of Salud Pública, Escuela Nacional de Estudios Superiores-León, Universidad Nacional Autónoma de México, León, Guanajuato, México

## Abstract

**Background:**

Oral candidiasis (OC) is the most common infection in patients wearing polymer-based dentures. Sustained-release systems for delivery (SRSDs) have been proposed as anti-OC therapeutics. Therefore, this systematic review aimed to determine which SRDS showed better antifungal results in in vivo models.

**Material and Methods:**

Electronic literature searches were conducted using PubMed/MEDLINE, Web of Science, and Google Scholar databases, limited to January 1, 1989, to May 31, 2025. The MeSH terms (PubMed) utilized included drug delivery systems, phytochemicals, plant extracts, herbal medicines, phytometabolites, antifungal agents, azoles, nystatin, macrolides, Candida spp., oral candidiasis, oral candidosis, Candida albicans, and Candida glabrata. Articles were included whose experimental design was in vivo or ex vivo models and whose objective was to determine the efficacy of sustained release systems as an anti-Candida treatment.

**Results:**

A total of 137 articles were retrieved and 125 were discarded because they did not match the principal objective of the study or did not meet the inclusion criteria. Twelve observational studies involving humans (adults aged 18 years) or animal models exposed to antifungal SRDSs were included. The most frequently used SRDSs were buccal mucoadhesive gel, mucoadhesive buccal tablets, and nanoparticles, while the most commonly used biomaterials were the bioadhesive polymers HPMC, NaCMC, carbopol 934, and sodium alginate. All antifungals incorporated in the SRDSs showed antifungal efficiency.

**Conclusions:**

Chitosan-coated bioadhesive polymers are the most promising options for treating OC.

## Introduction

Oral candidiasis (OC) is the most prevalent infection of the oral mucosa (1). Candida albicans has been identified as the principal etiological agent of OC, although clinical events related to non-albicans species are becoming increasingly frequent (2). C. albicans is a commensal yeast that switches to a pathogen condition under local or systemic conditions. The most common local condition that promotes OC is wearing polymer-based dentures, particularly polymethylmethacrylate (PMMA) dentures (3). Approximately 90% of denture wearers present with at least one clinical event of erythematous candidiasis (denture stomatitis) (2 - 4). Other populations at risk for OC are immunocompromised patients. Immunodeficiency is a principal risk factor for severe or deep mucosal and systemic fungal infections; therefore, it is closely related to an increase in morbidity and mortality related to Candida infections (5 , 6). Immunodeficient subjects at risk of oral candidiasis are numerous and of special interest, including people living with AIDS (PLWA) (1 , 2). Most of the available antifungals have been developed to prevent colonization or biofilm formation, and to eliminate and even eradicate infections caused by Candida species. The antimycotic arsenal with proven efficiency includes amphotericin B, clotrimazole, flucytosine, miconazole, nystatin, itraconazole, and ketoconazole, as well as phytometabolites with antimycotic effects (7). However, antimycotic drugs have disadvantages, including side effects, high risk of emergence of resistant strains, and low therapeutic potential, all of which limit their clinical use. Failure of antimycotic therapy is a possible event resulting from continuous, prolonged, or repetitive treatment, increasing the presence of Candida strains resistant to antifungal drugs. In the case of the oral cavity, anatomical and physiological conditions limit the antimycotic treatment or antimycotic effects. The dilution of the concentration levels of drugs due to saliva (8), the continuous swallow that minimizes the amount of available drug, and the superficial tension of the oral mucosa that does not allow adhesion of the drugs for a long time produces a low substantivity of the active agents in the oral environment, which affects their clinical performance. In such a way, several attempts to develop a local antifungal sustained-delivery system have been made (9 - 12). Since oral mycotic infections are chronic, long-term pharmaceutical treatments are necessary. Therefore, the duration of the active agent in the oral cavity is an essential parameter for the prevention and treatment of oral infections. Developing sustained-release delivery systems (SRDSs) for local applications may offer a promising strategy to overcome such problems. Among others, mucoadhesive formulations (12), wafers (13), nanoparticles (14), and polymer-based oral sustained-release varnishes (SRV) (15) have been developed mainly using in vitro assays (16). The next step is to conduct in vivo assays and clinical trials to assess the usefulness of SRDSs in antimycotic treatment. Unfortunately, the data from in vivo studies that proved the efficacy of SRDSs were dispersed. Therefore, this study aimed to systematically review the literature on antifungal SRDSs using in vivo models. Creating a systematic review of the existing scientific literature presents an excellent opportunity to refine the quality of future research and standardize research methods for this issue.

## Material and Methods

The present article is a systematic review focusing on antifungal SRDSs for treating OC. The study followed the Preferred Reporting Items for Systematic Reviews and Meta-Analyses (PRISMA) guidelines. This systematic review was conducted using the PECO format. Articles that met the following PECO criteria were included: P (population) of any animal model, and in the case of human studies that had been conducted in adults aged 18 years old, E (exposure) to sustained-release delivery system that contained an antimycotic, C (comparison) was not considered, and O (outcome) related to anticandidal inhibition or sensitivity of Candida cultures. Search strategy. The authors performed a systematic library search using electronic databases including MEDLINE, Web of Science, and Google Scholar. The time frame of the search was from January 1, 1989, to May 31, 2025. Additionally, a manual search was carried out on all bibliographies of the included papers. The keywords used were obtained from the MeSH terms (PubMed), including drug delivery systems, phytochemicals, plant extract herbal medicines, phytometabolites, antifungal agents, azoles, nystatin, and macrolides. Other keywords were Candida spp., oral candidiasis, oral candidosis, Candida albicans, and Candida glabrata. The research strategy was as follows (((DRUG DELIVERY SYSTEM) AND ((((ANTIFUNGAL AGENTS) OR (AZOLES)) OR (NYSTATIN)) OR (MACROLIDES))) AND (IN VIVO)) AND ((((ORAL CANDIDIASIS) OR (ORAL CANDIDOSIS)) OR (CANDIDA ALBICANS)) OR (CANDIDA GLABRATA)). EndNote X9.3.3 (Clarivate Analytics, Philadelphia, PA, USA) was used to compile the identified citations and eliminate duplicates. Subsequently, citations were uploaded for screening purposes. The titles and abstracts of each article were independently evaluated by two authors (LAGC and MCVV). A study was excluded if both reviewers believed it was unsuitable based on the titles and abstracts. The full text was read to reach a final decision in case of any uncertainty. Inclusion and exclusion criteria. All articles fulfilling the following eligibility criteria were included: 1) categorized as an observational study, 2) in case of human participant studies conducted on adults aged 18 years, in case of animal models, it was necessary to exhibit bioethical committee agreement; and 3) featured exposure to any type of antifungal sustained-release delivery system. Studies were excluded if they 1) assessed the oral health of children only; 2) consisted of case reports, commentaries, systematic reviews and meta-analyses, conference abstracts, letters, and editorials; 3) exclusively used in vitro models; 4) were written in any language other than English; and 4) did not meet the overall eligibility criteria. No restrictions were made on selecting studies based on sex, geographical location, or publication date (1989-2025). Data extraction. The following data were collected independently by each author from all the included studies: author, year, antifungal drug probed, type of sustained-release delivery system, experimental model, Candida species tested, and results. The research protocol was authorized by Comisión de Ética e Investigación (Ethical and Research Committee), Dental School, National Autonomous University of Mexico (number CIE0311112023).

## Results

**Figure 1:**
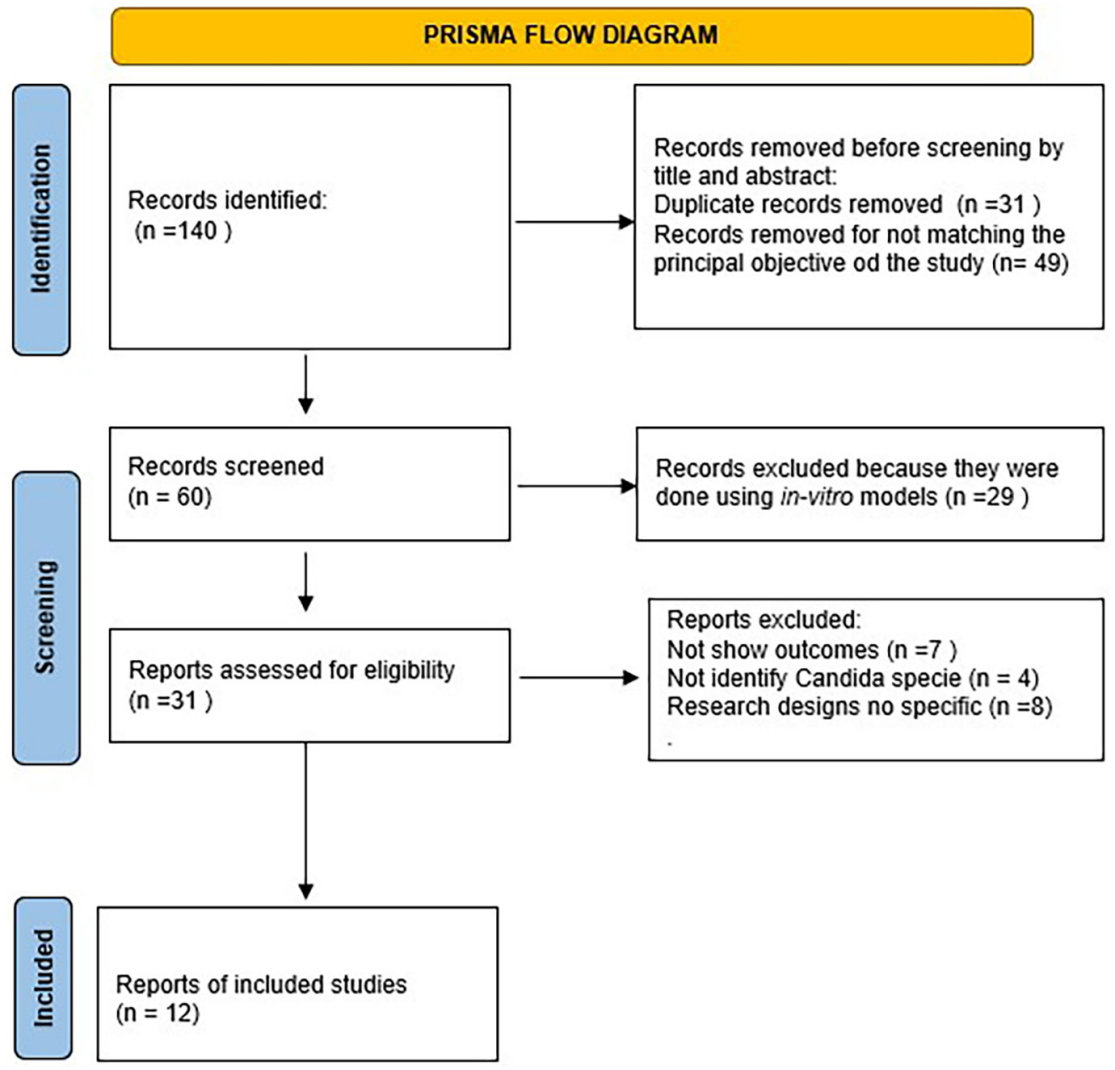
PRISMA flowchart result of research strategy.

A total of 140 articles were retrieved in the initial search. All were screened by title and abstract, and 80 were discharged to avoid matching the study's principal objective. Of the 60 articles remaining, 29 were excluded because they did not meet the inclusion criteria for the experimental model. The 31 remaining articles were assessed for eligibility, and 12 articles met all the inclusion criteria and were therefore included in the review. The remaining 19 articles were excluded due to the study design (Fig. 1).

1

The most frequently used drug delivery systems were buccal mucoadhesive gel, mucoadhesive buccal tablets, and nanoparticles. The most common biomaterials used are bioadhesive polymers, including hydroxypropyl methylcellulose (HPMC) (12 , 17), NaCMC (17), Carbopol 934 (11 , 17 , 18), and sodium alginate (17 , 19). Bioadhesive gels (11 , 12 , 18) and tablets (9 , 17 , 20) show efficient time adhesion, independent of the bioadhesive polymers or a combination of polymers used. The characteristics of mucoadhesive nanoparticles were improved by adding a coating of chitosan (16 , 21) and buco-adhesive films (19). The most frequently tested antimycotic drugs are polyenes, including Amphotericin B (20) and nystatin (9 , 21), and azoles, including miconazole (17) and fluconazole (16). In addition, two natural plant products were tested: eugenol (22) and P. granatum extract (18). Independent of their source, natural or artificial, all antifungals incorporated into SRDSs showed anticandidal efficiency. Analysis of the included articles showed the diversity of the animal models employed, including rats (20 , 22), mice (12), rabbits (11 , 16), moths (23), and pigs (21). The most frequently used in vivo model is that of humans (17 - 19 , 25). However, only one human study was conducted in subjects with OC related to prosthesis use. This study evaluated the antifungal properties of an extract of the P. granatum oral gel and a commercial miconazole oral gel. Although the miconazole oral gel showed slightly better clinical results, individuals treated with P. granatum did not have side effects. Three other human studies have investigated the adhesive properties of drug delivery systems. The most conspicuous characteristics of each study are shown in Table 1.

Table 1

## Discussion

A systematic review examined whether in vivo models of antimycotic SRDS were successful. Studies have shown that antifungal SRDSs possess the characteristics needed for primary therapeutic targets, and all studies reported inhibitory effects on Candida species regardless of the antifungal tested. This is the first systematic review of the in vivo effects of oral antifungal drug delivery systems. Two essential objectives drive oral SDRS design: 1) ensuring sufficient contact time of antifungal drugs with oral mucosa for inhibitory effect and 2) maintaining the minimum inhibitory concentration (MIC) of the antifungal. These objectives require overcoming obstacles, such as continuous swallowing and saliva dilution. This review shows that buco-adhesive systems (9 , 12 , 17 - 21) are the most common. Human studies have used mucoadhesive gels (18), tablets (9 , 17), or films (19), with gels and tablets showing the most promise. Regarding MIC, human clinical studies or animal models have not shown whether this aim was achieved. Although not explained by the authors, technical difficulties could be the reason, since controlled environments, such as in vitro protocols, show that antimicrobials in drug delivery systems reach MIC. Therefore, in vivo research protocols are required to assess the MICs of antifungals in SDRSs. Adding a chitosan coating to nanoparticles (16 , 21) improves therapeutic results (16 , 19 , 21), suggesting that drug combinations could enhance antifungal effects. Phytometabolites can be used as alternatives. Studies tested natural products: Garg A, Singh S, et al. (22) used eugenol, while Vasconcelos LC, et al. (18) tested P. granatum extract. Eugenol (22) showed a higher antimycotic efficiency than P. granatum (18). However, a comparison could not be made because the eugenol study used a nanoparticle-coated lipid system (22) on a rat model, while the P. granatum study (18) was a human study using a buco-adhesive tablet. Developing ex vivo or in vivo models using established antimycotic phytometabolites (26 - 28) in SDRSs is necessary. The limited side effects, availability, and cost of plant extracts, especially those from P. granatum (27), make them attractive for future clinical use. The development of a clinically successful SDRS antifungal agent will affect the quality of life of many individuals. Two populations could benefit: PLWA and acrylic muco-supported denture wearers. Currently, 38 million PLWAs exist worldwide, indicating that the HIV pandemic remains uncontrolled (24). About 70-80% of patients develop OC during their lifetime (25). These 38 million potential patients suffer or will suffer from OC, making antimycotic SDRS urgently needed. Among denture wearers, 11-70% have prosthetic stomatitis (4 , 29 , 30), with Candida carriers exceeding 88% (31). Polymethyl methacrylate (PMMA) is the main material used for muco-supported prostheses (3 , 4). PMMA prosthesis bases harbor bacterial plaques and fungi (32), with Candida albicans being the most frequent in the palatal mucosa and denture base (4). Yeasts adhere to denture surface irregularities and proliferate through co-aggregation with oral bacteria including Streptococcus sanguis, S. gordonii, S. oralis, S. anginosus, Staphylococci, Actinomycetes, and Lactobacilli (32). Mature C. albicans biofilms form a complex network of yeast, pseudohyphal, and hyphal cells embedded in a protective extracellular matrix (33). A cannabidiol-based varnish showed 90% biofilm reduction in an ex vivo tooth model (34), suggesting the potential use of plant extracts in varnishes as SRDS for denture bases. In elderly or senile patients, poor manual hygiene skills lead to prosthetic stomatitis. Two solutions were proposed: adding antifungals to acrylic (35) and using mucoadhesive SDRSs, both of which showed promising results. Human studies were conducted in healthy subjects aged 22-40 years (9 , 17 - 18), making the results inapplicable to the elderly. Research protocols involving older adults with dentures with or without prosthetic stomatitis are needed.

## Conclusions

There are a wide variety of antifungal agents with proven anti-Candida action. Thus, the challenge is to determine which SDRS is the most suitable, least expensive, and easiest to handle for geriatric patients, which maintains a consistently high concentration of the antifungal rather than the antifungal itself. In this sense, the results of this systematic review suggest that the development of bioadhesive polymers coated with chitosan is the most promising option.

## Figures and Tables

**Table 1:** Summary of the characteristics of the articles included in the systematic review.

Author	Antifungal	Sustained-release delivery system	Experimental model	Candida species	Result
Garg A, Singh S (2011) [22]	Eugenol	Loaded solid lipid nanoparticles	Rats	C. albicans	Eugenol concentration is maintained for an extended time at the site of action. Improved antifungal efficacy of eugenol
Abou Samra MM, et al. (2020) [11]	Ciclopirox olamine (CPO)	CPO- loadedhybridized vesicular (HV) system (Buccal Gels). The gel was formed using cholesterol, lipoidS45 or phospholipoid 90H, and Carbopol	Male albino rabbits	C. albicans	CPO-loaded HV gel is more active therapeutically, with greater inhibition of C. albicans
Zhou L, et al. (2017) [20]	Amphotericin B(AmB)	Mucoadhesive buccal tablet of AmB/ monomethoxy poly(ethylene glycol)-poly (epsilon caprolactone) - graft-polyethylenimine (MPP micelles)	Sprague Dawley rats. In-vitro antifungal properties	C. albicans	MPP micelles present enhanced antifungal activity against the biofilm state of C. albicans. The AmB/MPP micelles could be candidates for an effective buccal delivery system
Kong EF, et al. (2015) [11]	Histatin-5	Bioadhesive hydrogels of hydroxypropyl methylcellulose (HPMC), plus Hst-5 at a concentration of 2 g/ml	Mice C57BL/6	C. albicans (SC5314)	No CFU of C. albicans were recovered from 46.3% of treated mice. The bioadhesive gel showed inhibition of the proliferation of colonizing C. albicans in-vivo
Borges AC, (2018) [36]	Cold atmospheric pressure plasmas	Amplitude-modulated cold atmospheric plasma jet device operating with Helium	Mice (Swiss)	C. albicans (ATCC 18804)	Amplituded-modulated cold atmospheric plasma was more effective than nystatin after 24 h in reducing tissue invasion, however, after 48 h the effect was similar
RenÃ§ber S, et al. (2016) [16]	Fluconazole	Mucoadhesive nanoparticle of acrylic and methacrylic acids or their esters, coated with chitosan	New Zealand white rabbits	C. albicans (ATTC 90028)	The local administration of buccal mucoadhesive chitosan coated NP charged with fluconazole achieved clinical success
MartÃ­n MJ, et al. (2015) [21]	Nystatin(Nys)	Mucoadhesive microspheres of alginate plus an anionic copolymer of 1,4 linked--d-mannuronic acid and -l-guluronic acid, coated with chitosan	Crossbred pigs ex-vivo assay of cheek mucosa of pigs. In-vitro antifungal properties	C. albicans	Mucoadhesive microspheres of alginate coated with chitosan produced inhibition of C. albicans. Chitosan provided a sustained release of Nys
Vasconcelos LC, et al. (2003) [18]	Extract of P. granatum	Oral mucoadhesive gel using Carbopol 940, triethanolamine and 0.5 ml of P. granatum extract	Humans with diagnosis of denture stomatitis (erythematous candidiasis)	C. albicans	P. granatum oral gel had slightly lesser clinical results than miconazole oral gel. Individuals treated with P. granatum no reported side effects
Mohammed and Khedr H (2003) [17]	Miconazole nitrate	Buccal bioadhesive tablet using the bioadhesive polymers HPMC, NaCMC, carbopol 934 (Cp), and sodium alginate, and containing miconazole nitrate	Healthy humans subjects (aged 22-40 years). in-vitro antifungal properties	C. albicans	The antifungal activity of miconazole buccal bioadhesive tablets was longer (>8 hr) and did not cause irritation, hindrance, or any side effects. Tablets prepared with NaCMC/Carbopol or sodium alginate/NaCMC were preferred by the volunteers
Juliano C, et al. (2008) [19]	Chlorhexidine	Mono- and double-layered buccoadhesive films made of alginate and/or hydroxypropylmethyl cellulose and/or chitosan	Healthy human(males only)	------	The alginate-chitosan buccoadhesive films were well tolerated and the salivary levels of chlorhexidine diacetate concentrations reached its highest level 120 min after the application
Llabot JM, et al. (2009) [9]	Nystatine	Mucoadhesive tablets compounded by carbomer (C), lyophilized carbomersodium salt (CNaL) and nystatine	Healthy humans (aged 20-30 years)	------	Mucoadhesive tablets remained attached to the buccal mucosa for 27 - 30 min. Mucoadhesive tablets caused light reversible irritation. Nystatine was several times higher than MIC22 over a period of approximately 4.5 h
Marena GD, et al. (2024) [23]	Micafungin (MICA)	MICA encapsulated in a sunflower oil and cholesterol nanoemulsion (NEM)	Galleria mellonellalarvae	C. auris (InP13, CBS10913, Kro 2, CBS 15603, SP96, SP94, VEN C6, BRA 2), C. albicans (ATCC 5314) and C. parapsilosis (ATCC 22019)	Encapsulation of MICA in a nanoemulsion enhances its antifungal activity against mature biofilms of C. auris and C. parapsilosis but not against C. albicans

1

## Data Availability

The datasets used and/or analyzed during the current study are available from the corresponding author.
